# Galvanic-Cell-Reaction-Driven Deposition of Large-Area Au Nanourchin Arrays for Surface-Enhanced Raman Scattering

**DOI:** 10.3390/nano8040265

**Published:** 2018-04-23

**Authors:** Zhongbo Li, Kexi Sun, Zhaofang Du, Bensong Chen, Xuan He

**Affiliations:** 1 College of Light-Textile Engineering and Art, Anhui Agricultural University, Hefei 230036, China; dzf@ahau.edu.cn; 2 Key Laboratory of Nanomaterials and Nanotechnology, Institute of Solid State Physics, Chinese Academy of Sciences, Hefei 230031, China; 3 College of Physics and Electronic Information, Luoyang Normal University, Luoyang 471022, China; kxsun@issp.ac.cn; 4 Institute of Chemical Materials, China Academy of Engineering Physics, Mianyang 621900, China; xuan.hellen@caep.cn

**Keywords:** surface-enhanced Raman scattering, galvanic cell reaction, Au nanourchins, PCBs

## Abstract

Here we report a low-cost synthetic approach for the direct fabrication of large-area Au nanourchin arrays on indium tin oxide (ITO) via a facile galvanic-cell-reaction-driven deposition in an aqueous solution of chloroauric acid and poly(vinyl pyrrolidone) (PVP). The homogeneous Au nanourchins are composed of abundant sharp nanotips, which can served as nanoantennas and increase the local electromagnetic field enhancement dramatically. Finite element theoretical calculations confirm the strong electromagnetic field can be created around the sharp nanotips and located in the nanogaps between adjacent tips of the Au nanourchins. In addition, the interparticle nanogaps between the neighboring Au nanourchins may create additional hotspots, which can induce the higher electromagnetic field intensity. By using rhodamine 6G as a test molecule, the large-area Au nanourchin arrays on ITO exhibit active, uniform, and reproducible surface-enhanced Raman scattering (SERS) effect. To trial their practical application, the Au nanourchin arrays are utilized as SERS substrates to detect 3,3’,4,4’-tetrachlorobiphenyl (PCB-77) one congener of polychlorinated biphenyls (PCBs) as a notorious class of persistent organic pollutants. The characteristic Raman peaks can be still identified when the concentration of PCB-77 is down to 5 × 10^−6^ M.

## 1. Introduction

Over the past decades, gold (Au) nanostructures have received widespread research interests, due to their fascinating size- and shape-dependent physicochemical properties [1,2,3,4,5,6], and their various promising applications in catalysis [7,8], chemical sensing [9,10,11,12], plasmonics [13], and surface-enhanced Raman scattering (SERS) [14,15,16,17,18,19,20,21,22]. In order to tune their unique properties and further improve their performance, Au nanostructures with diverse geometric features, including plate [23], cube [24], cage [25], wire [26], sphere [27], decahedron [28], and branched nanostructures [29] have been synthesized through a rich variety of chemical methods. Recently, urchin-like Au nanostructures, such as Au nanostars [18,30] and Au nanourchins [31,32,33,34,35,36,37], a class of nanostructures with sharp protrusions, have aroused great interest, because of the strong relation between the fantastic morphology and their unique optical properties. The individual nanourchin is composed of many sharp nanotips serving as nanoantennas, where localized surface plasmons (LSPs) can be excited by the incident light to enhance the local electromagnetic (EM) field intensity by 2–5 orders of magnitude [35]. Therefore, it can be expected that the chemical inert Au nanourchins can be used as reliable SERS-active substrates for real-time and label-free detection of ultra-trace chemicals [32,38].

To obtain these three-dimensional (3D) extraordinary Au nanourchins, routine methods are usually involved colloidal strategy where diverse reduction agents and surfactants are used, such as l-dopa [32,37] and ascorbic acid [36], forming individual Au urchin structure in a liquid solution, which is beneficial to colloidal research. However, Au nanourchins achieved by the colloidal strategy are not the optimal option for wafer-based applications, including optics, electrocatalysis, chemical/biochemical sensing, and SERS substrates. On the other hand, large area Au nanourchin array, based wafer substrates could be produced via a two-step process combining a variety of prefabricated 3D templates, such as polyaniline (PANI) membrane [34], polystyrene (PS) nanospheres [33], Ag-nanohemisphere arrays [39] and then assembling or electrodepositing Au nanoparticles onto the 3D templates through electrochemical systems [40,41], which broaden application fields of the Au nanourchins. However, it is usually time-consuming and expensive to fabricate large-scale 3D Au nanourchin array-based wafer substrates. Thus, simple and low-cost approaches to large-scale homogeneous Au nanourchin arrays with sufficient sharp nanoantennas are still highly demanded for SERS-based sensitive detection.

**Figure 1:**
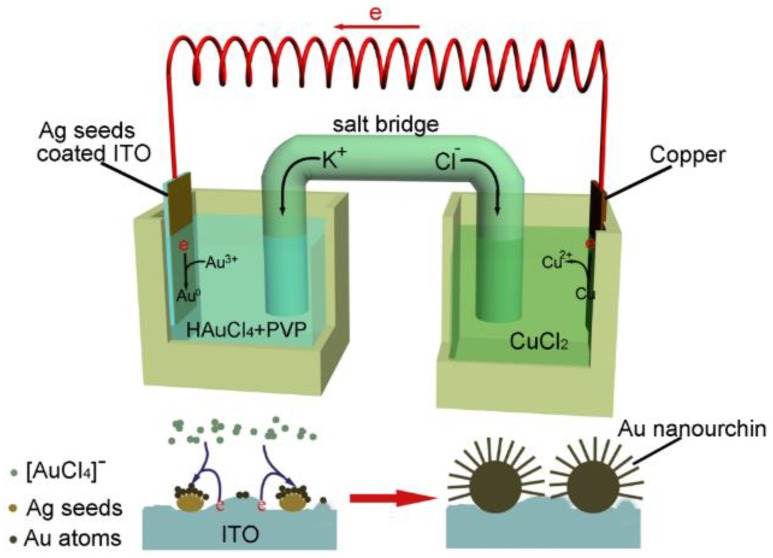
Schematic illustration for the formation of Au nanourchin arrays on indium tin oxide (ITO) through galvanic-cell-reaction-driven deposition.

Here, we present an effective and low-cost galvanic-cell-reaction-driven deposition approach to directly synthesize large-area Au nanourchin arrays, with each Au nanourchin grafted with abundant sharp nanotips on indium tin oxide (ITO) wafer. As shown in Figure 1, two half-cells connected with a salt-bridge and wire constitute a complete galvanic cell. The right half-cell consists of a piece of copper foil which is immersed into a solution of CuCl_2_, and the left half-cell consists of an ITO substrate and a mixed electrolyte. As the standard reduction potential of [AuCl_4_]^−^/Au pair (1.498 V vs. standard hydrogen electrode, SHE) on the ITO side is higher than that of the Cu^2+^/Cu pair (0.337 V vs. SHE), the copper foil therefore serves as the anode, where the oxidation of copper releases electrons which are transferred to the ITO side along the wire, while ITO serves as the cathode on which [AuCl_4_]^−^ ions are attracted and then reduced into Au^0^. The galvanic-cell-induced Au^0^ atoms or clusters on the ITO substrate would preferentially nucleate at the precoated Ag seeds via heterogeneous nucleation, and then the nuclei grow into Au nanourchins under the drive of the galvanic-cell-reaction. Meanwhile, potassium ions travel along the salt bridge to the left half-cell and chloridions travel along the salt bridge to the right half-cell to balance the charges of the solutions, and thereby allow the reaction. As Au nanourchins are composed of abundant sharp nanotips and nanogaps between the neighboring nanotips which can induce strong local EM field enhancement, Au nanourchin arrays on the ITO exhibit high SERS sensitivity showing promising potential applications in the fields of chemical sensing, surface-enhanced Raman scattering, and so forth. The presented approach is cost-effective and simple in operation, which can be also used to fabricate large-area homogeneous metal nanostructure arrays with different shapes and sizes.

## 2. Materials and Methods

### 2.1. Chemicals

Chloroauric acid, silver nitrate, copper chloride, agar, sodium borohydride, rhodamine 6G (R6G), poly(vinyl pyrrolidone) (PVP, K30), and copper foils were purchased from Shanghai Aladdin Reagent Co. Ltd. (Shanghai, China). All of the chemicals were used without further purification.

### 2.2. The Preparation of Salt-Bridge

In a typical preparation process, 3 g agar powder was added to 97 mL deionized (DI) water. Then, the mixed solution was heated and stirred until the complete dissolution of the agar powder. Then, 30 g KCl was added quickly into hot agar solution and fully stirred. Finally, the hot mixed solution was casted into U-shaped glass tube and cooled at room temperature for 12 h.

### 2.3. The Synthesis of Ag Seeds Colloidal Solution

In a typical synthetic process, 0.005 g AgNO_3_ and 0.04 g of poly(vinyl pyrrolidone) (PVP) were added to 80 mL DI water. The mixed solution was stirred for several minutes, then 20 mL of an aqueous 5 × 10^−4^ M NaBH_4_ solution which had been aged at room temperature for 2 h was added quickly, then stirred for 1 h and aged at room temperature for 24 h.

### 2.4. Coating Ag Seeds on ITO Substrate

Firstly, a piece of ITO glass (3 cm × 1 cm) was rinsed with acetone, ethanol, and DI water respectively. After drying in high-purity flowing nitrogen, 200 μL of as-prepared Ag colloidal solution was casted onto a clean ITO glass fixed on the spin coater. The rotating speed was kept at 1500 rounds/min for 3 min. This process was repeated for 1~3 times.

### 2.5. Synthesis of Au Nanourchin Arrays on Ag Seeds Spin-Coated ITO Substrates

First, the copper foil and the Ag seeds spin-coated ITO glass were connected with a conducting wire. Then, the ITO glass was dipped into 20 mL mixed aqueous solution of HAuCl_4_ (1 g/L) and PVP (20 g/L), and a piece of copper foil (1.5 cm × 1.5 cm) was dipped into 80 mL 0.5 g/L CuCl_2_ aqueous solution. A U-shaped KCl salt bridge was dipped into the two half-cells for deposition. After the deposition for 5 h at room temperature, the ITO glass with the products was then taken out, cleaned with DI water several times, and dried with high-purity flowing nitrogen.

### 2.6. Characterizations

The resultant products were characterized by using X-ray diffraction (XRD) (Philips X’pert-PRO, PANalytical, Almelo, The Netherlands), scanning electronic microscope (SEM, sirion 200, Thermo Fisher Scientific, Hillsboro, OR, USA) transmission electron microscope (TEM, JEOL 2010, JEOL Ltd., Tokyo, Japan). Absorption spectra of as-prepared substrates were recorded using a UV3600, MPC-3100 spectrometer (Shimadzu, Kyoto, Japan).

### 2.7. Raman Measurements

A confocal microprobe Raman system (Renishaw, inVia, Gloucestershire, UK) was used to acquire Raman spectra with a laser beam of 633 nm wavelength, 5 mW power. For the comparison of the SERS activity of different substrates, the SERS substrates achieved with different concentrations of PVP and ion sputtering Au-nanoparticle film were immersed in 1 mL 10^−5^ M R6G solutions, then taken out, rinsed with DI water and air-dried. To evaluate the SERS sensitivity toward R6G, the optimal Au nanourchin arrays were chosen as the SERS substrates and immersed in R6G solution with different concentration (10^−6^, 10^−8^, 10^−9^ M) for 3 h, then taken out, rinsed with DI water, and air-dried. For the SERS detection of polychlorinated biphenyls (PCBs), the PCB-77 was dissolved in the *n*-hexane, and small pieces of optimal Au nanourchin arrays were immersed in different concentrations of PCB-77 solution for about 7 h. Then, the SERS substrates were taken out and dried in the fuming cupboard. To demonstrate the uniformity of SERS signal of the prepared substrates, mapping Raman and collecting spectra at different points were carried out using the optimal Au nanourchin arrays. Raman mappings of “hotspots” over the optimal Au nanourchin arrays were performed on Raman imaging system (Renishaw, inVia). R6G (10^−5^ M) was used as molecular probe. A 633 nm laser was scanned over the substrates and Raman spectra were collected as a function of position. When the Raman images were generated from the SERS intensity of the 611 cm^−1^ band of R6G, the incident laser power was kept constantly at 5 mW with the step size of 2 μm and the exposure time was 1 s. Renishaw WiRE 3.4 software (Renishaw, Gloucestershire, UK) was used as data acquisition and control. To reveal the reproducibility of SERS signal of optimal SERS substrates, the optimal Au nanourchin array substrates from five batches were immersed in 1 mL 10^−5^ M R6G solutions, then taken out, rinsed with DI water, and air-dried. And the relative standard deviation (RSD) of the average SERS intensities of the five Au nanourchin array substrates from different batches was calculated.

### 2.8. FEM Calculations

A finite element method was used to study the electromagnetic field distribution which was conducted using a reported method [42]. The geometry dimensions of the Au nanourchin applied in the model were taken from the scan electron microscopy and transmission electron microscopy observations, and had a diameter of ~650 nm, and a nanotip length of ~150 nm. A dense mesh was used to giving an excellent accuracy with the maximum element size are less than 6 nm for the ball, and less than 3 nm for the nanotips. The boundary condition was perfect electric conductor for the Nanourchin and scattering boundary conditions for the air. The optical constant of Au (εAu = −10.4 + 1.2i) at the wavelength of 633 nm was taken from the literature [43,44]. 

## 3. Results and Discussion

**Figure 2:**
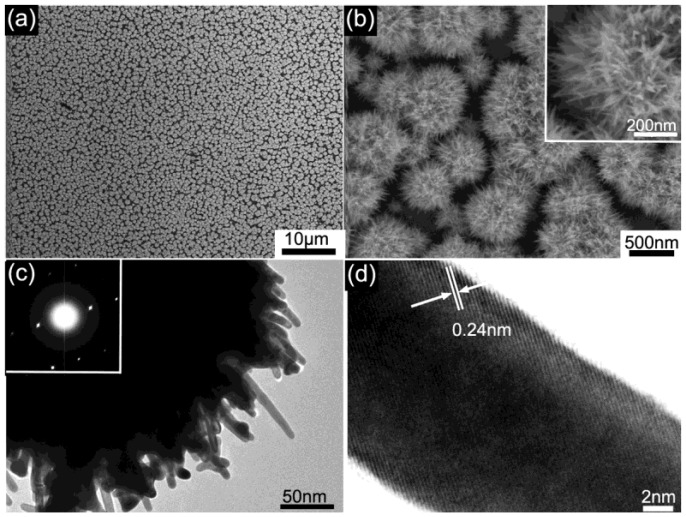
(**a**,**b**) Different magnified SEM images of Au nanourchin arrays on ITO; (**c**)TEM image of a single Au nanourchin; the inset in (**c**) is SAED pattern from one nanotip on the Au nanourchin; (**d**) HRTEM image of a single Au nanotip.

After the galvanic-cell-reaction-driven deposition had proceeded for 5 h, large-scale homogeneous Au nanourchin arrays are achieved on the ITO wafer, as shown in the top-view scanning electron microscope (SEM) images (Figure 2a,b). The enlarged-view SEM image (Figure 2b) reveals that flocky Au nanourchins with an average diameter of ~650 nm are uniformly deposited on the ITO. It is worth mentioning that Au nanourchins are composed of high density sharp nanotips (around 300~400 tips on each particle) and nanogaps (between the adjacent nanotips). Furthermore, the needle-like nanotips of the Au nanourchin are confirmed by the close-up SEM view (inset of Figure 2b) with an average diameter of ~20 nm. Transmission electron microscopy (TEM) and selected area electron diffraction (SAED) provide detailed structural information of the prepared Au nanourchins. A TEM image shown in Figure 2c displays that individual Au nanourchins contain a number of sharp and protruding branches, varying from 60 to 150 nm. The SAED pattern in the inset of Figure 2c and lattice spacing in the high-resolution TEM (HR-TEM) shown in Figure 2d verify that the individual nanotip has a single-crystalline nature. The lattice spacing is estimated to be 0.24 nm, which is in good accordance with the distance of the (111) crystal plane of the face-centered cubic (fcc) Au crystals, demonstrating that the growth of the Au nanotips preferentially occurs in the (111) directions. Energy dispersive X-ray spectroscopy (EDS) (Figure S1) obtained on Au nanourchins confirms the formation of Au. The as-prepared Au nanourchin arrays are further investigated using X-ray diffraction to determine crystalline structure. The typical XRD pattern of the as-prepared Au nanourchin arrays is shown in Figure S2, revealing four characteristic diffraction peaks at 38.3°, 44.3°, 64.3°, and 77.6°, which can be indexed as (111), (200), (220), and (311) crystal planes of face-centered cubic (FCC) Au, which correspond with standard data (JCPDS 04-0784). It is worth noting that the intensity ratio (4.4) of the (111)/(200) diffraction peak in the XRD spectrum is larger than that (1.9) of the standard diffraction peak of Au powder, suggesting that the as-deposited Au nanourchin arrays abound in (111) crystalline planes in the Au nanourchins [45].

**Figure 3:**
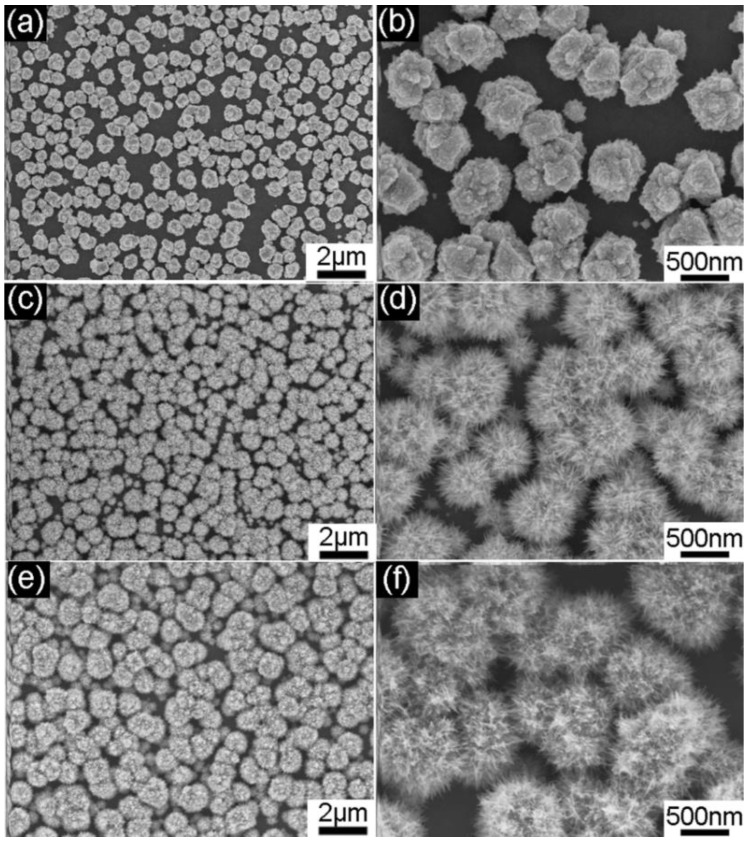
SEM images of the as-deposited products achieved on ITO spin-coated with (**a**,**b**) 0 μL Ag colloidal solution; (**c**,**d**) 200 μL Ag colloidal solution; (**e**,**f**) 200 μL Ag colloidal solution for three times. The concentration of PVP is kept at 20 g/L.

In order to reveal the growth mechanism of the Au nanourchin arrays, the influence of Ag seeds and the concentration of polyvinylpyrrolidone (PVP) on the formation of the Au nanourchin arrays was investigated. It has been demonstrated that Ag seeds play an important role in the growth of the Au nanourchins [32,37]. Our experiments also verify that Ag seed pre-coating is a critical step for obtaining good-quality Au nanourchin arrays. If the commercial ITO substrates were not coated with any Ag seeds, the as-deposited products consisting of a large number of flower-like gold particles with an average diameter of 700 nm (Figure 3a,b) were achieved, and each of the flower-like gold particles comprises a lot of irregular particles (Figure 3b). Once Ag seeds (200 μL colloidal solution, Figure S3) were spin-coated on the ITO for once in advance, a number of individual Au nanourchins (with each particle consisting of bundles of nanoneedles) could be obtained (Figure 3c,d). If more Ag seeds (200 μL colloidal solution three times) were spin-coated onto the ITO substrate, a larger number of individual Au nanourchins could be achieved in high yield (Figure 3e,f). The more the Ag seeds (corresponding to the more spin-coating times) coated on the ITO substrates in advance, the higher the density of the Au nanourchin on the ITO substrates. In addition, the absorption spectra of the Au nanostructures achieved under the different conditions reveal that distinct surface plasmon resonance (SPR) positions could be generated (Figure S4). The absorption spectrum of the nanourchin arrays prepared with three rounds of spin-coating reveals strong plasmon absorption from 550 to 800 nm with an absorption band peak centered at 675 nm, indicating that the Au nanourchin arrays can generate high electromagnetic enhancement of Raman signals when the excitation laser with a wavelength of 633 nm was used in Raman measurements.

**Figure 4:**
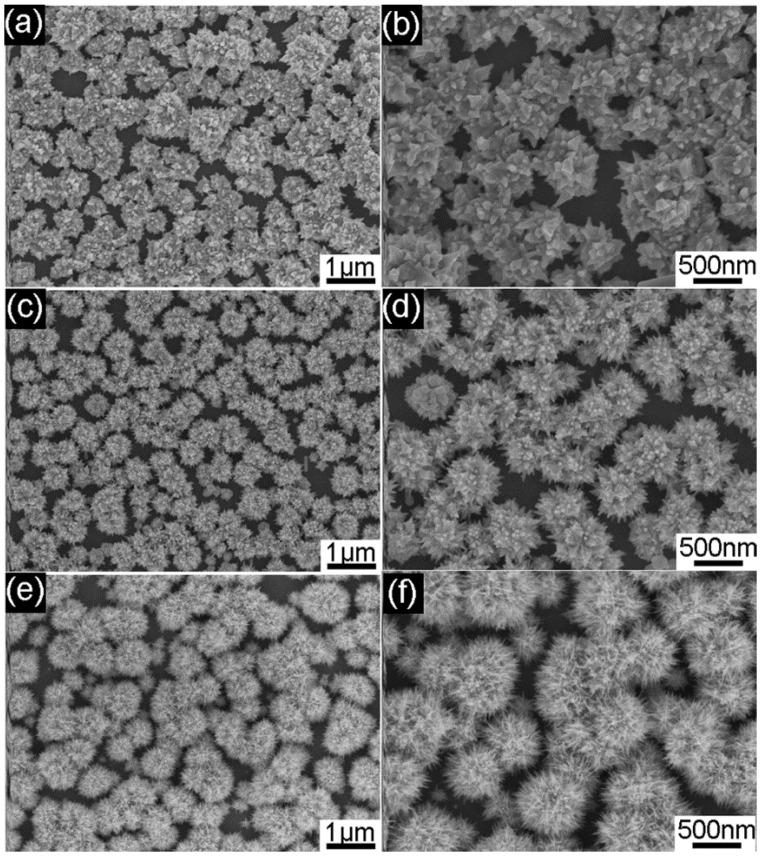
SEM images of the as-deposited products achieved at different concentrations of PVP: (**a**,**b**) 2 g/L; (**c**,**d**) 10 g/L; (**e**,**f**) 20 g/L. All the ITO substrates were spin-coated with 200 μL colloidal solution once.

Importantly, it is found that the PVP concentration also has a great impact on the growth of the Au nanourchin. Figure 4 shows the Au nanourchin arrays prepared under the PVP concentration of 2, 10, and 20 g/L, respectively. When the concentration of PVP in the electrolyte is low (i.e., 0~2 g/L), urchin-like Au nanostructures with sparse nanocones can be deposited onto the surface of the ITO, as shown in Figure 4a,b. With the increase of PVP concentration from 2 to 10g/L, the number of the nanoprotruding blocks on the urchin-like Au nanostructures increase gradually (Figure 4c), and the size of individual Au nanourchin on the ITO becomes smaller, as shown in Figure 4d. If the concentration of PVP is further increased to 20 g/L, Au nanourchins with dense slender nanoneedles in the arrays are achieved (Figure 4e,f). The number of the individual Au nanourchin increase gradually as the concentration of PVP increases. 

From the aforementioned results, it can be seen that the morphology of urchin-like Au nanostructures achieved on ITO can be regulated by controlling the amount of the Ag seeds and PVP via the galvanic-cell-reaction-driven growth. A possible crystal growth mechanism could be proposed. Once Ag seed-coated ITO is immersed in the solution simultaneously, galvanic replacement between Ag seeds and [AuCl_4_]^−^ ions occurs, and Ag seeds are eroded, therefore, some specific sites can be created on the surface of Ag seeds. Then, a U-shaped KCl salt bridge is dipped into the two half-cells simultaneously, galvanic-cell-reaction starts to occur, and two important influences are produced in this galvanic-cell-reaction-driven deposition system [46,47]. Firstly, the reduction of [AuCl_4_]^−^ into Au^+^ by the galvanic-cell-reaction significantly decreases the rate of galvanic replacement, suppressing the formation of porous structures. Secondly, the mild reducing speed of galvanic-cell-reaction can also lead to the reduction of Au^+^ into Au^0^, which tend to attach and nucleate at the specific sites (small projections or tips) of Ag seeds (seeded growth) [46]. It has been proven that a galvanic replacement between Ag seeds and [AuCl_4_]^−^ ion-generated specific sites (small projections or tips) on Ag seeds can induce the growth of branched Au nanostructures [32,37]. As the galvanic-cell-reaction proceeds, overgrowth dominates, and galvanic-cell-reaction generated Au^0^ atoms are deposited on these specific sites continuously. Meanwhile, PVP acts as dispersing agent. With the increase of PVP concentration, the amount of nucleation centers increases. As the reaction continues, more Au atoms are generated and continuously attached to the surface of the nucleation centers, inducing the preferential growth of multiple nanotips. Consequently, the number of the Au nanourchin and Au nanotips increases, and the diameter of the tips decreases.

**Figure 5:**
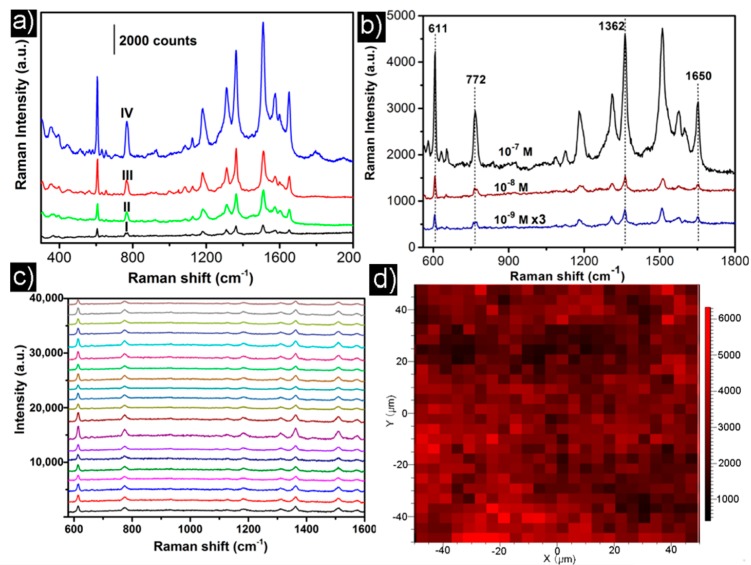
(**a**) The SERS spectra of R6G collected on ion sputtering Au-nanoparticle film (I) and the Au nanourchin arrays achieved at different concentrations of PVP: (II) 2 g/L, (III) 10 g/L, (IV) 20 g/L. Data integration time: 10 s; (**b**) SERS spectra collected on the optimal Au nanourchin arrays (Figure 4e) exposed to different concentrations of R6G aqueous solution data integration time: 10 s; (**c**) SERS spectra of 10^−6^ M R6G at different locations of the optimal Au nanourchin arrays shown in Figure 4e. data integration time: 5 s; (**d**) Mapping for optimal Au nanourchin arrays acquired with a step of 2 μm.

As distinctive Au nanourchins consisting of sharp nanotips can induce an intense SERS effect, the SERS performance of the Au nanourchin arrays was evaluated by using rhodamine 6G (R6G) molecule as Raman probe. Figure 5a shows the SERS spectra of 10^−5^ M R6G collected on the different Au nanoparticles achieved on ITO wafers with the different experimental conditions. As shown in Figure 5a, it is noteworthy that all the urchin-like Au nanourchin arrays achieved with different PVP concentrations exhibit higher SERS activity than the ion sputtering Au-nanoparticle film. This result demonstrated that the 3D Au nanourchin arrays can support structurally enhanced SERS activity, which mainly stems from the higher SERS electromagnetic field induced by the sharp nanotips and high density of hotspots located in the nanogaps between the adjacent nanotips. With an increase of PVP concentration from 2 to 20g/L, it is quite obvious that the SERS signal is improved. As the PVP concentration increases, the ever-increasing SERS activity can further demonstrate that higher density of nanotips on the Au nanourchins and higher density of hotspots located in the nanogaps between the adjacent nanotips support intense local electromagnetic field and show a large contribution to the SERS enhancement. Au nanourchin arrays achieved with 20 g/L PVP which show the highest SERS activity were employed as the optimal SERS substrates, to further reveal the sensibility toward R6G. Figure 5b shows the spectra of R6G with different concentrations (10^−6^, 10^−8^, and 10^−9^ M) on the optimal Au nanourchin arrays. It can be seen that three distinct characteristic peaks of R6G at 611 cm^−1^ (C–C–C ring in-plane bending mode), 772 cm^−1^ (C–H out of plane bending mode), and 1362 cm^−1^ (aromatic C–C stretching vibration mode) are revealed, respectively [48,49,50]. As shown in shown in Figure 5b, the distinct characteristic peaks can be still recognized in the Raman spectra even at the low concentration of 10^−9^ M, demonstrating high SERS sensibility. 

The uniformity and reproducibility of SERS-signal of the optimal Au nanourchin arrays were further examined using the optimal Au nanourchin arrays as substrates. In order to study uniformity of the optimal substrates, collecting spectra randomly at different points and mapping Raman were carried out. The SERS spectra of R6G (Figure 5c) collected at 20 spots on the optimal substrate demonstrate that the average relative standard deviation of the intensities for the peak at 611 cm^−1^ is less than 15.3%, indicating that the SERS substrate shows a good signal uniformity. In order to further verify the signal uniformity of the as-prepared substrates, mapping Raman was performed on an area of 100 × 100 μm^2^ on optimal Au nanourchin arrays. The corresponding mapping for the optimal arrays acquired with a step of 2 μm and presented as a color-coded integrated intensity of the characteristic peak of R6G at 611 cm^−1^ was shown in Figure 5d. It can be seen that the “hotspots” over the optimal Au nanourchin arrays are uniform. The good signal uniformity is attributed to the homogeneous and high-density hotspots created by the high density of 3D Au nanourchin arrays. The RSD of the average SERS intensities of the five Au nanourchin array substrates from different batches (Table S1 in the Supplementary Materials) was 9.7%, confirming good substrate-to-substrate reproducibility of SERS signal.

**Figure 6:**
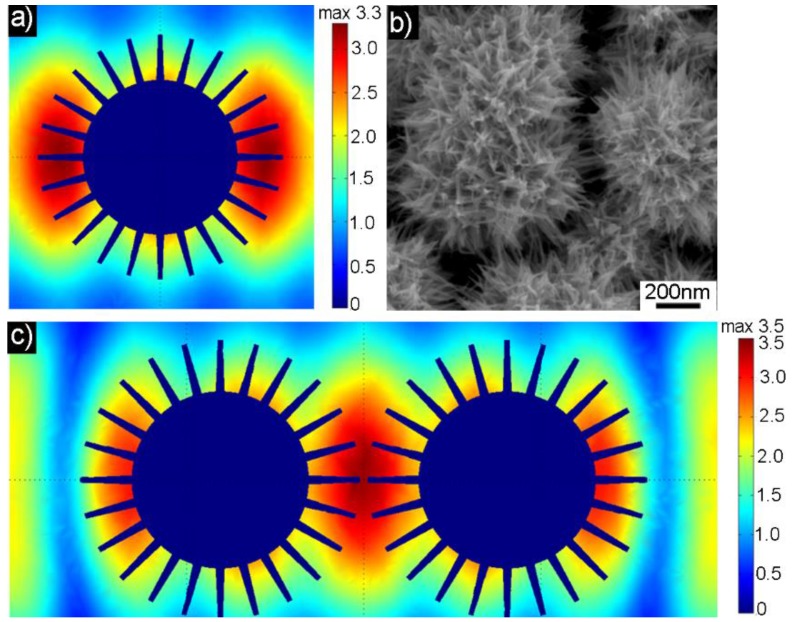
(**a**) Calculated electromagnetic field distribution and intensity of a single Au nanourchin shown in Figure 2a at 633 nm wavelength; (**b**) The enlarged SEM image of Au nanourchins closing to each other (**c**) Electromagnetic field distribution of two neighboring Au nanourchin.

To further verify the structural enhancement of the Au nanourchin arrays, a finite element method was used for calculations. The geometry dimensions of the Au nanourchin applied in the model were taken from the scan electron microscopy and transmission electron microscopy observations, and had a diameter of ~650 nm, and a nanotip length of ~150 nm. Figure 6a is the calculated electromagnetic field distribution of the individual Au nanourchin at the 633 nm incident laser wavelengths. The simulation result demonstrates that the maximal electromagnetic field or hotspots can be created around the sharp nanotips, and located in the nanogaps between adjacent tips of the Au nanourchins. In addition, the interparticle effect between the neighboring Au nanourchins (Figure 6b) was also investigated (Figure 6c), where the distance of the nanotips between the two neighboring Au nanourchins is 20 nm. It can be seen that the plasmon coupling could be achieved according to the electromagnetic field distribution, which can induce the additional hotspots between the two neighboring Au nanourchins and be beneficial to the improvement of SERS signals. Furthermore, the electromagnetic field coupling effect between the Au nanourchins is very useful to improve the uniformity of SERS signals.

**Figure 7:**
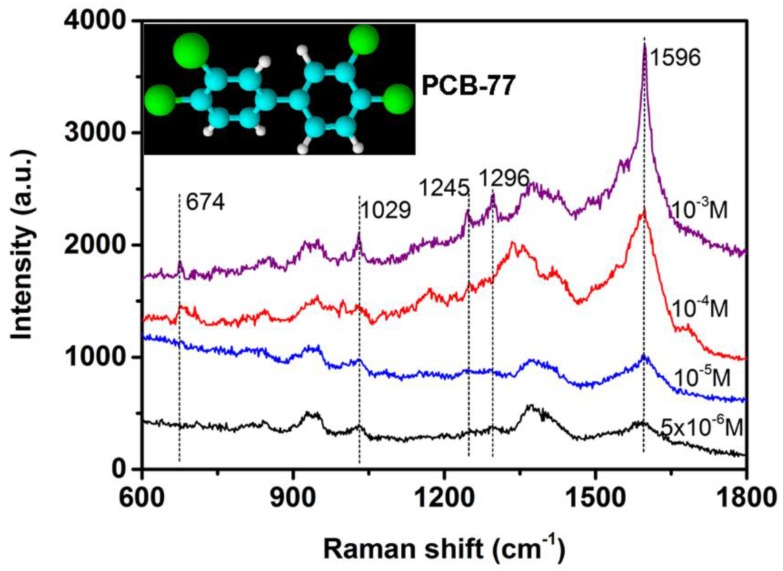
SERS spectra collected on the optimal Au nanourchin arrays (Figure 4e) exposed to different concentrations of PCB-77 solution. Data integration time: 30 s.

To trial their potential practical applications of the as-prepared 3D Au nanourchin arrays, the optimal Au nanourchin arrays were utilized as SERS substrates to detect PCB-77, one congener of polychlorinated biphenyls (PCBs). Figure 7 shows the SERS spectra of PCB-77 with different concentrations (10^−3^, 10^−4^, 10^−5^, 5 × 10^−6^ M) dispersed on the optimal substrate. Five fingerprint characteristic peaks at 674, 1029, 1245,1296, and 1596 cm^−1^ in the SERS spectra (Figure 7) correspond well to the reported characteristic peaks [16,51,52,53], and can still be identified when the concentration of PCB-77 is down to 5 × 10^−6^ M. Therefore, the as-prepared the Au nanourchin arrays can serve as effective SERS-substrates for the detection of toxic organic pollutants in the environment.

## 4. Conclusions

In summary, the generation of large-scale homogeneous Au nanourchin arrays have been achieved directly on ITO wafers via a simple galvanic-cell-reaction-driven deposition approach. Ag seeds on the ITO and the concentration of PVP in the mixed aqueous solution are crucial to obtaining monodispersed Au nanourchins consisting of abundant sharp nanotips. As strong electromagnetic fields can be created around the sharp nanotips and in the nanogaps between the adjacent nanotips on the same or neighboring Au nanourchins, the Au nanourchin arrays on ITO exhibit excellent SERS performance and SERS-signal reproducibility. Using the Au nanourchin arrays as SERS substrates, the characteristic peaks of PCB-77 can still be identified when the concentration of PCB-77 is down to 5×10^−6^ M, demonstrating great potential in SERS-based organic pollutant detection.

## Supplementary Materials

The following are available online at http://www.mdpi.com/2079-4991/8/4/265/s1, Figure S1: EDS from Au nanourchin arrays shown in Figure 2b; Figure S2: The XRD pattern of the as-prepared sample shown in Figure 2b; Figure S3: TEM image of the pre-prepared Ag colloidal particles; Figure S4: Absorption spectra of Au nanostructures achieved under the different experimental conditions. Table S1: The average 611 cm^−1^ peak intensities of R6G SERS spectra collected from 5 substrates of different batches.
