# Supplementary material for: Galvanic-Cell-Reaction-Driven Deposition of Large-Area Au Nanourchin Arrays for Surface-Enhanced Raman Scattering
Source: Nanomaterials (Basel). 2018 Apr 23;8(4):265. doi: 10.3390/nano8040265 (PMC5923595; doi:10.3390/nano8040265)
Supplement: Supplementary file 1 [file nanomaterials-08-00265-s001.pdf]

## Supplementary Material

# Galvanic-Cell-Reaction-Driven Deposition of Large-Area Au Nanourchin Arrays for Surface-Enhanced Raman Scattering

**Zhongbo Li [1,2,*], Kexi Sun [3], Zhaofang Du [1], Bensong Chen [2,*] and Xuan He [4]**

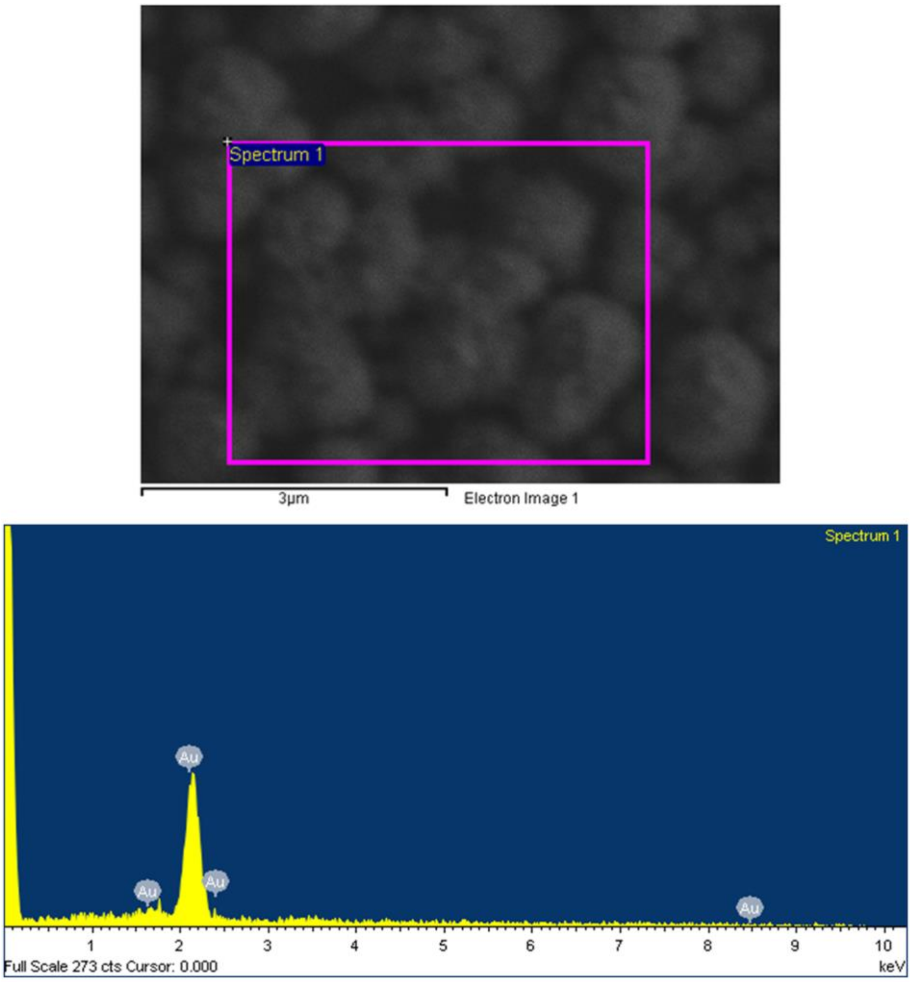


**Figure S1.** EDS from Au nanourchin arrays shown in Figure 2b.

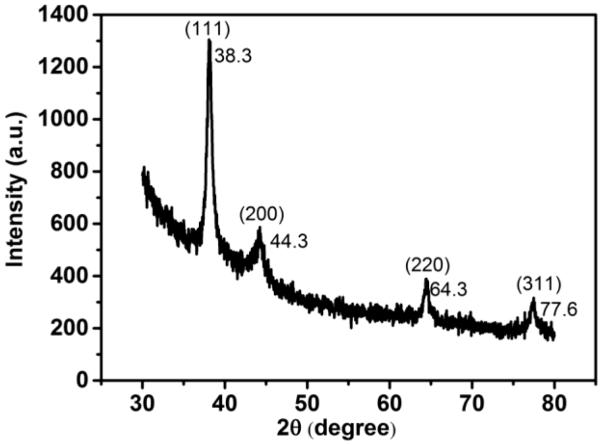


**Figure S2.** The XRD pattern of the as-prepared sample shown in Figure 2b.

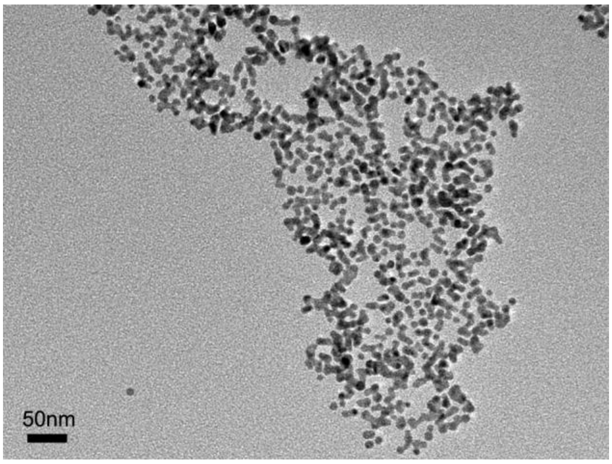


**Figure S3.** TEM image of the pre-prepared Ag colloidal particles.

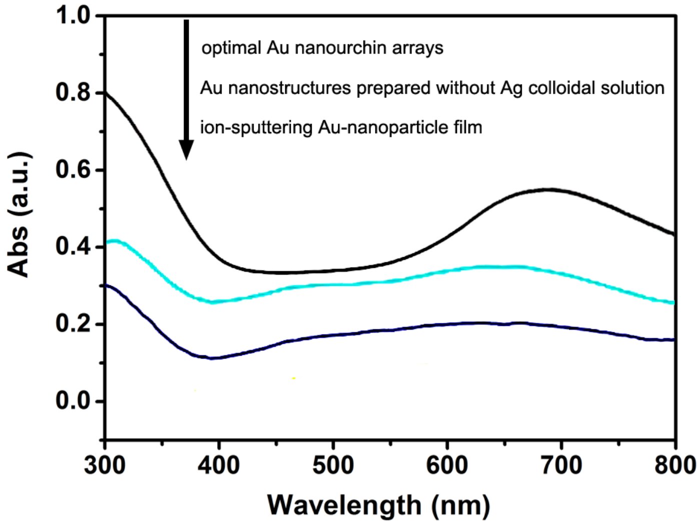


**Figure S4.** Absorption spectra of Au nanostructures achieved under the different experimental conditions.

**Table S1.** The average 611 $cm^{-1}$ peak intensities of R6G SERS spectra collected from 5 substrates of different batches.

| Substrate Number | 1 | 2 | 3 | 4 | 5 |
|---|---|---|---|---|---|
| Raman Intensity | 5263 ±606 | 5120 ±525 | 6101 ±701 | 4789 ±423 | 5780 ±646 |
